# Aerosol-Administered Adelmidrol Attenuates Lung Inflammation in a Murine Model of Acute Lung Injury

**DOI:** 10.3390/biom12091308

**Published:** 2022-09-16

**Authors:** Livia Interdonato, Ramona D’amico, Marika Cordaro, Rosalba Siracusa, Roberta Fusco, Alessio Filippo Peritore, Enrico Gugliandolo, Rosalia Crupi, Stefano Coaccioli, Tiziana Genovese, Daniela Impellizzeri, Rosanna Di Paola, Salvatore Cuzzocrea

**Affiliations:** 1 Department of Chemical, Biological, Pharmaceutical and Environmental Sciences, University of Messina, 98166 Messina, Italy; 2 Department of Biomedical, Dental and Morphological and Functional Imaging, University of Messina, Via Consolare Valeria, 98125 Messina, Italy; 3 Department of Veterinary Science, University of Messina, 98168 Messina, Italy; 4 General Medical Clinic and Medical Therapy, Rheumatology and Medical Therapy of the Pain, University of Perugia, “Polo di Terni”, “AO Santa Maria” of Terni, 06129 Perugia, Italy

**Keywords:** acute lung injury, Adelmidrol, mast cells, inflammation, oxidative stress

## Abstract

Acute lung injury (ALI) is a common and devastating clinical disorder with a high mortality rate and no specific therapy. The pathophysiology of ALI is characterized by increased alveolar/capillary permeability, lung inflammation, oxidative stress and structural damage to lung tissues, which can progress to acute respiratory distress syndrome (ARDS). Adelmidrol (ADM), an analogue of palmitoylethanolamide (PEA), is known for its anti-inflammatory and antioxidant functions, which are mainly due to down-modulating mast cells (MCs) and promoting endogenous antioxidant defense. The aim of this study is to evaluate the protective effects of ADM in a mice model of ALI, induced by intratracheal administration of lipopolysaccharide (LPS) at the dose of 5 mg/kg. ADM 2% was administered by aerosol 1 and 6 h after LPS instillation. In this study, we clearly demonstrated that ADM reduced lung damage and airway infiltration induced by LPS instillation. At the same time, ADM counteracted the increase in MC number and the expression of specific markers of MC activation, i.e., chymase and tryptase. Moreover, ADM reduced oxidative stress by upregulating antioxidant enzymes as well as modulating the Nf-kB pathway and the resulting pro-inflammatory cytokine release. These results suggest that ADM could be a potential candidate in the management of ALI.

## 1. Introduction

Acute lung injury (ALI) and acute respiratory distress syndrome (ARDS), two acute inflammatory conditions, are a major cause of respiratory failure and one of the most challenging clinical conditions with significant morbidity and mortality [1]. ALI is characterized by alteration of the endothelium and alveolar epithelial barrier, resulting in increased microvascular permeability, pulmonary edema, and polymorphonuclear neutrophil infiltration, all of which contribute to decreased respiratory function [2]. Evidence has proposed that several pathophysiological pathways are activated during ALI, especially during the early phase of the disease [3,4], in which inflammatory response plays a key role [1]. ALI can be modeled in rodents by the administration of LPS through tracheal instillation [5,6,7,8]. Local administration of LPS causes an acute and vigorous migration of inflammatory cells into the lung tissue, leading to the overproduction of pro-inflammatory cytokines, including interleukin (IL)-6, IL-1β and tumor necrosis factor (TNF)-α [9,10]. Among the inflammatory cells, mast cells (MCs) stand out for their involvement in the pathophysiology of ALI [11]. In particular, MCs activation induce the release of the contents of their granules, including specific proteases such as chymase and tryptase that contribute to the progression of inflammatory diseases on the respiratory system [12,13]. Additionally, several studies support the role of oxidants and oxidative stress in the pathogenesis of ALI [14,15,16]. In the context of ALI/ARDS, there are many potential sources of reactive oxygen species (ROS), including leukocytes (neutrophils, monocytes, and macrophages), parenchymal cells (endothelial and epithelial cells, fibroblasts, and myocytes) and circulating oxidant-generating enzymes [16]. Excessive ROS production generated by the injured endothelium/epithelium, as well as recruited leukocytes, amplifies the tissue damage and pulmonary edema [17,18]. Thus, a cross-link between inflammatory response and oxidative stress is involved in the development of ALI [19,20]. Therefore, new approaches are needed to improve the clinical outcomes of the patients affected with the disease. In this regard, we investigated the properties of Adelmidrol (ADM), a palmitoylethanolamide (PEA) analogue that belongs to the ALIAmide family (Autacoid Local Injury Antagonist Amides) [21]. It is well known that ADM has important anti-inflammatory properties due to the regulation of MC activation [22,23,24]. Recently, it has also been shown that ADM is able to boost endogenous antioxidant defense [25], indirectly enhancing its protective function. Therefore, the aim of this study is to evaluate the beneficial effects of ADM in an LPS-induced ALI model, through the modulation of inflammatory and oxidative pathways.

## 2. Materials and Methods

### 2.1. Animals

Male CD1 mice (25–30 g, Envigo, Milan, Italy) were housed in a controlled environment, with food and water ad libitum. The University of Messina Review Board for animal care (OPBA) approved the study (ethical protocol code: 266/2021-PR). All in vivo experiments followed the new directives of the USA, Europe, Italy, and the ARRIVE guidelines.

### 2.2. Induction of Acute Lung Injury

For intratracheal (i.t.) instillation, animals were anesthetized with isoflurane (2%), and LPS was instilled as previously described [26,27]. Briefly, a 1 cm long ventral midline cervical incision was used to expose the trachea, and LPS was injected using a bent 27-gauge tuberculin needle. Escherichia coli LPS (026: B6L3755, Sigma Aldrich, St. Louis, MO, USA) was administered by a single i.t. instillation at the dose of 5 mg/kg suspended in saline solution (total volume = 0.05 mL per animal) [1]. Sham animals were subjected to the same procedure but received saline instead of LPS. ADM 2% in isotonic solution was administered by aerosol, with a Lovelace nebulizer (In-Tox Products, Albuquerque, NM, USA) being used to create an atmosphere in an exposure chamber (Research and Consulting Co., AG, Basel, Switzerland), as previously described by D’Amico et al. [28].

### 2.3. Experimental Groups

Mice were randomized into the following experimental groups (n = 12/group):

- LPS group: Mice received LPS i.t. and were treated with the vehicle (saline);
- LPS + ADM group: Mice received LPS i.t. and were treated with ADM 2% aerosol 1 h and 6 h after LPS instillation;
- Sham group: Similar to LPS group, but mice received saline i.t. instead of LPS;
- Sham + ADM: Mice received saline i.t. and were treated with ADM 2% aerosol 1 h and 6 h after saline instillation (data not shown, as no significant difference was ever observed between Sham and Sham + ADM).

At 24 h after induction, all animals were sacrificed, and bronchoalveolar lavage fluid (BALF) as well as lung tissues were collected for further analysis.

### 2.4. Proteins Concentration and Cell Counts in BALF

The cell count in BALF was carried out as previously described [1]. Briefly, BALF was collected by cannulating the trachea and lavaging the lung twice with 0.7 mL of phosphate-buffered saline (PBS) [1]. The washing solution were removed by aspiration and BALF was centrifugated at 800 rpm [29]. The supernatant was stored at −20 °C, while the pelleted cells were resuspended in PBS. Then, the total cells in BALF were enumerated by counting with a hemocytometer in the presence of the trypan blue stain. For differential cell counting, Wright’s Giemsa stain was performed, and the leukocyte and macrophage populations present in BALF were counted. After staining, the differential count was carried out by the standard morphological protocol under a light microscope [30]. To determine the protein concentration and to measure the pro-inflammatory cytokines, the supernatants in BALF were analyzed by a BCA Protein Assay Kit (ThermoFisher, 00161, Rome, Italy. while the levels of IL-6 (#DKW12-2060; Dakewe Biotech Co., Ltd., Bensheim, Germany), IL-1β (#MBS8800273; Biosource International, Camarillo, CA, USA) and TNF-α (#30907; BioLegend, San Diego, CA, USA)) were detected using ELISA [1,31].

### 2.5. Measurement of Lung Edema

At the end of experiment, wet lung weights were recorded. The lungs were subsequently dried for 48 h at 80 °C and weighed again. The water content in the lung tissues was calculated as the ratio of wet/dry weight of the lung [32].

### 2.6. Histological Examination

Lung sections were stained with Hematoxylin and Eosin (H&E) for histological analysis [33,34,35,36] and with toluidine blue to determine MC degranulation [37]. Every section was examined using a Leica DM6 microscope; (Leica Microsystems SpA, Milan, Italy) associated with Leica LAS X Navigator software (Leica Microsystems SpA, Milan, Italy). Every slide was viewed at a magnification of 10× and morphological changes were evaluated by two blinded investigators [38,39,40,41]. Lung injury score was measured according to the methods reported previously [42,43]. The criteria are as follows: 0 = no damage, l = mild damage, 2 = moderate damage, 3 = severe damage, 4 = very severe histologic changes.

### 2.7. Myeloperoxidase (MPO) Assay

The MPO activity was measured as previously described [44,45,46] and represented in units per gram of wet tissue weight, defined as the amount of enzyme capable of decomposing 1 μmol of peroxide per minute at 37 °C.

### 2.8. Immunohistochemical Localization of Chymase and Tryptase

Immunohistochemical analysis was performed as previously described [47,48,49]. Primary antibodies anti-MC chymase (1:100, Santa Cruz Biotechnology (SCB) Heidelberg, Germany, #sc59586) and anti-MC tryptase (1:100, SCB, #sc59587) were incubated overnight on the lung tissue sections. Images were collected using a Leica DM6 microscope; a 10× magnification is shown (Leica Microsystems SpA, Milan, Italy) following a typical procedure [50,51,52,53]. The positive pixel intensity value obtained was connected to the histogram profile [54,55].

### 2.9. Measurement of Oxidative Stress

The malondialdehyde (MDA; #A003-1-2, Nanjing, China), glutathione (GSH; #A006-2-1, Nanjing, China) and catalase (CAT; #A007-1-1, Nanjing, China) levels in the lung tissues were measured using activity assay kits (Nanjing Jiancheng Bioengineering Institute) [52,56,57,58].

### 2.10. Analysis of Western Blots

Western blots were performed on lung samples as described in our previous studies [59,60,61]. The following antibodies were used: anti-IkBα (1:1000, SCB, #sc1643), anti-NF-kB p65 (1:1000; SCB, #sc8414), anti-Nrf2 (1:5000; SCB, #sc365949), anti-HO-1 (1:5000; SCB, #sc136960), MnSOD (1:5000 SCB #sc137254), anti-β-actin (1:5000; SCB, #sc8432) and anti-lamin A/C antibody (1:5000; Sigma-Aldrich, St. Louis, MO, USA). The membranes were then incubated with IgG peroxidase-conjugated secondary antibody-conjugated bovine mouse IgG or IgG peroxidase-conjugated goat anti-rabbit (1:2000, Jackson ImmunoResearch, Baltimore, MD, USA) [58,62,63,64]. Protein expression was quantified by densitometry with BIORAD ChemiDocTM XRS + software and normalized to housekeeping genes β-actin and lamin A/C as previously reported [64,65]. Images of blot signals were imported to analysis software (Image Quant TL, v2003, Rome, Italy.) [41,60].

### 2.11. Materials

Unless, otherwise stated, all compounds used in this study were purchased from Sigma-Aldrich Company Ltd. (Milan, Italy). ADM was obtained from Epitech Group SpA.

### 2.12. Statistical Evaluation

All values are expressed as mean ± standard error of the mean (SEM) of N observations. The images shown are representative of the last three experiments performed on diverse experimental days on tissue sections collected from all animals in each group. For in vivo studies, N represents the number of animals used. The results were analyzed by one-way ANOVA followed by a Bonferroni post hoc test for multiple comparisons. A *p* value less than 0.05 was considered significant.

## 3. Results

### 3.1. ADM 2% Aerosol on Histopathological Analysis and Neutrophil Activity

**Figure 1:**
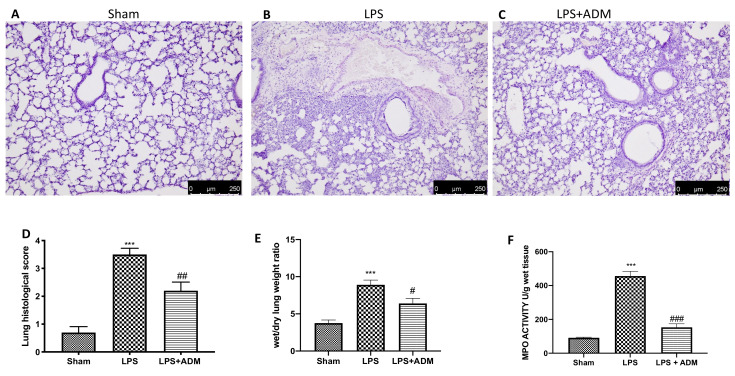
Histological analysis: sham (**A**), LPS (**B**), LPS + ADM 2% (**C**). Histological score (**D**). Wet/dry lung weight ratio (**E**). MPO activity (**F**). A 10× magnification is shown. Data are expressed as the mean ± SEM of N = 6 mice/group. *** *p* < 0.001 vs. sham; # *p* < 0.05 vs. LPS; ## *p* < 0.01 vs. LPS; ### *p* < 0.001 vs. LPS.

First, we analyzed the ADM effects on histopathological damage, including alveolar congestion, bleeding, neutrophil infiltration and thickness of alveolar wall/hyaline membrane formation. H&E exhibited extensive tissue damage and extracellular matrix deposition in the lungs of LPS-treated animals (Figure 1B,D) compared to the sham groups (Figure 1A,D). Aerosol treatment with ADM 2% significantly minimized lung damage (Figure 1C,D). We also evaluated the presence of lung edema by the ratio of wet/dry weight of the lung and neutrophil infiltration by the MPO assay. The ratio of wet/dry weight of the lung and MPO activity were increased by i.t. injection of LPS, while ADM 2% significantly reduced both parameters. (Figure 1E,F).

### 3.2. ADM 2% Aerosol on Inflammatory Cells and Pro-Inflammatory Cytokines in BALF

**Figure 2:**
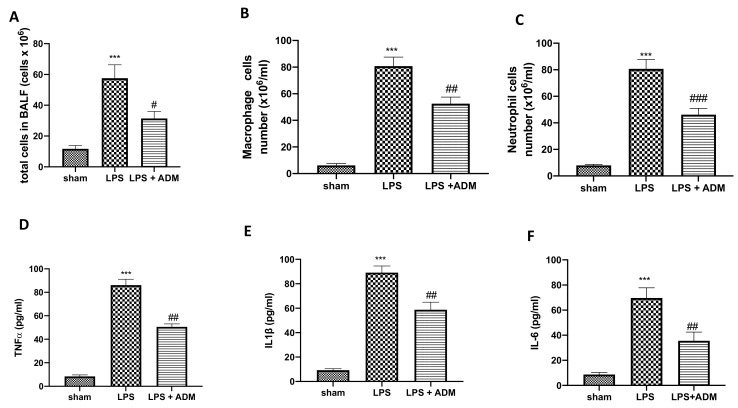
Cell infiltration expression in BALF. Total cell number (**A**), Macrophages (**B**), Neutrophils (**C**). Expression of proinflammatory cytokine: TNF-α (**D**), IL-1β (**E**), IL-6 (**F**). Data are expressed as the mean ± SEM of N = 6 mice/group. *** *p* < 0.001 vs. sham; # *p* < 0.05 vs. LPS; ## *p* < 0.01 vs. LPS; ### *p* < 0.001 vs. LPS.

To determine whether ADM was able to reduce cell infiltration, we measured inflammatory cell counts in the BALF 24 h after LPS i.t. instillation. We found a substantial increase in total cell counts (Figure 2A), macrophages (Figure 2B) and neutrophils (Figure 2C) in BALF taken from LPS-treated animals compared to Sham mice. The number of inflammatory cells in BALF was significantly reduced after ADM 2% aerosol (Figure 2A–C). Additionally, we examined BALF levels of the pro-inflammatory cytokines. TNF-α (Figure 2D), IL-1β (Figure 2E) and IL-6 (Figure 2F) levels were significantly increased in the LPS group compared to Sham mice. On the contrary, cytokines release in BALF was markedly reduced in mice treated with ADM (Figure 2D–F).

### 3.3. ADM 2% Aerosol on MC Number

**Figure 3:**
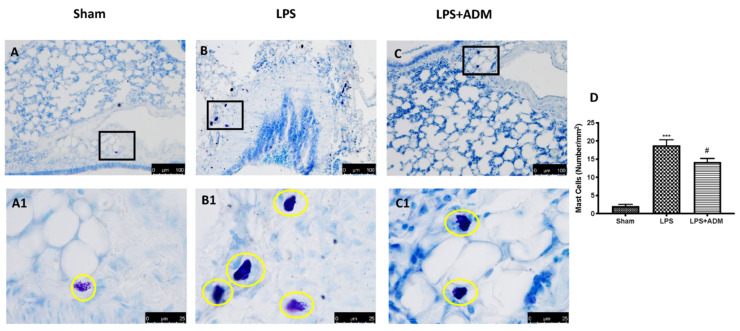
Mast cells indicated by toluidine blue staining: Sham (**A**), LPS (**B**), LPS + ADM 2% (**C**), mast cell count (**D**). A 20× and 100× magnification is shown. Data are expressed as the mean ± SEM of N = 6 mice/group. *** *p* < 0.001 vs. sham; # *p* < 0.05 vs. LPS.

Toluidine blue staining of lung sections was used to assess the MC number. We detected a higher number of MCs in the LPS group (Figure 3B,B1,D), compared to Sham animals (Figure 3A,A1,D). ADM 2% aerosol reduced in a significant manner MC hyperplasia in lung tissues (Figure 3C,C1,D).

### 3.4. ADM 2% Aerosol on Chymase and Tryptase Expression

**Figure 4:**
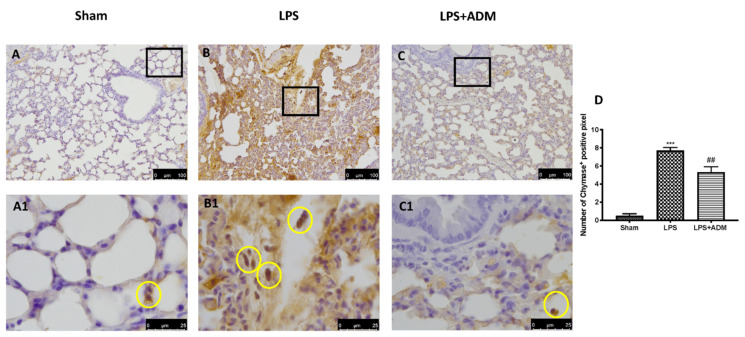
Immunohistochemical analysis for chymase: Sham (**A**,**A1**), LPS (**B**,**B1**), LPS + ADM 2% (**C**,**C1**), graphical quantification (**D**). A 20× and 100× magnification is shown. Data are expressed as the mean ± SEM of N = 6 mice/group. *** *p* < 0.001 vs. sham; ## *p* < 0.01 vs. LPS.

**Figure 5:**
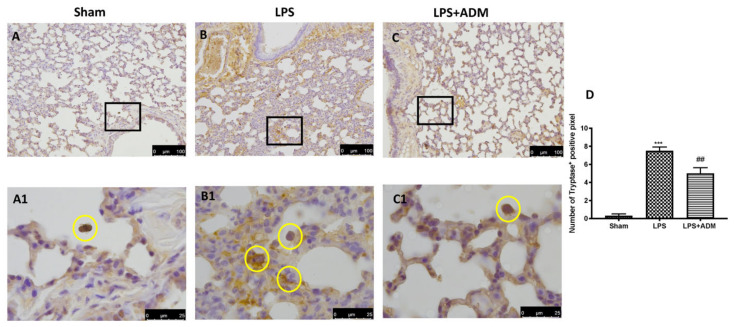
Immunohistochemical analysis for tryptase: Sham (**A**,**A1**), LPS (**B**,**B1**), LPS + ADM 2% (**C**,**C1**), graphical quantification (**D**). A 20× and 100× magnification is shown. Data are expressed as the mean ± SEM of N = 6 mice/group. *** *p* < 0.001 vs. sham; ## *p* < 0.01 vs. LPS.

To confirm the activity of MCs and their activation, we evaluated the chymase and tryptase expressions by immunohistochemical analysis. LPS instillation enhanced chymase activity in the lungs (Figure 4B,B1,D), compared to Sham mice (Figure 4A,A1,D). At the same way, LPS increased tryptase expression in the lungs (Figure 5B,B1,D) compared to Sham mice (Figure 5A,A1,D). ADM 2% aerosol was able to reduce both preformed mediators expression (Figure 4C,C1,D for chymase; Figure 5C,C1,D for tryptase).

### 3.5. ADM 2% Aerosol on Oxidative Stress

**Figure 6:**
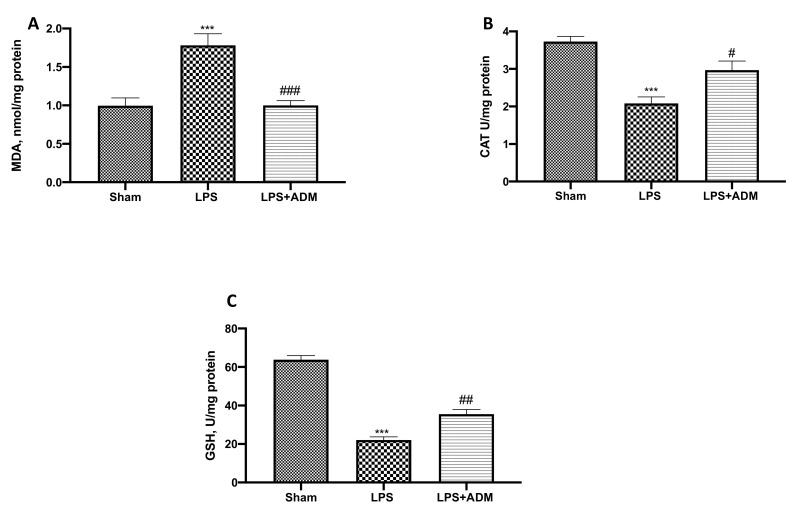
Markers of oxidative stress: MDA (**A**), CAT (**B**), and GSH (**C**). Data are expressed as the mean ± SEM of N = 6 mice/group. *** *p* < 0.001 vs. sham; # *p* < 0.05 vs. LPS; ## *p* < 0.01 vs. LPS; ### *p* < 0.001 vs. LPS.

To evaluate the effect of ADM on oxidative stress, we performed MDA activity as an indicator of lipid peroxidation. LPS-treated mice showed increased MDA levels, while the LPS + ADM group showed lower levels of MDA (Figure 6A). Additionally, we investigated CAT and GSH levels for the oxidative response. LPS induced an important decrease in CAT (Figure 6B) and GSH (Figure 6C) levels, compared to the Sham groups. Both levels of antioxidant indicators were markedly increased by ADM 2% aerosol treatment.

### 3.6. ADM 2% Aerosol on Nrf2 Pathway

**Figure 7:**
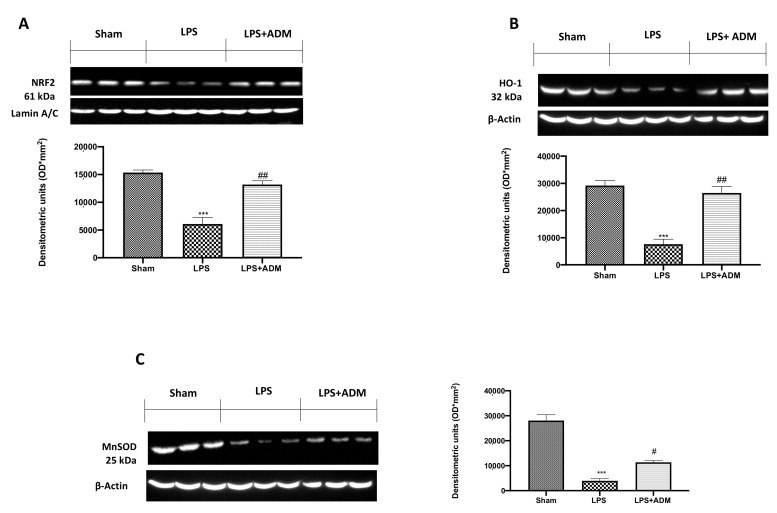
Western blot analysis for: Nrf2 (**A**); HO-1 (**B**); MnSOD (**C**). A demonstrative blot of lysates with a densitometric analysis for all animals is shown. Data are expressed as the mean ± SEM of N = 6 mice/group. *** *p* < 0.001 vs. sham; # *p* < 0.05 vs. LPS; ## *p* < 0.01 vs. LPS.

To confirm the antioxidant effect of ADM, we evaluated the Nrf2 pathway by Western blot analysis. Our results showed an important reduction in Nrf2 expression in the vehicle group compared to the sham group, while ADM was able to upregulate Nrf2 expression (Figure 7A). Consequently, we evaluated HO-1 (Figure 7B) and MnSOD (Figure 7C) expression, which are regulated by the Nrf-2 pathway. Our results showed an important decrease in HO-1 and MnSOD expression in the LPS group, compared to sham mice; on the contrary, ADM partially restored the expression of both endogenous enzymes (Figure 7B,C).

### 3.7. ADM 2% Aerosol on Inflammatory Pathway

**Figure 8:**
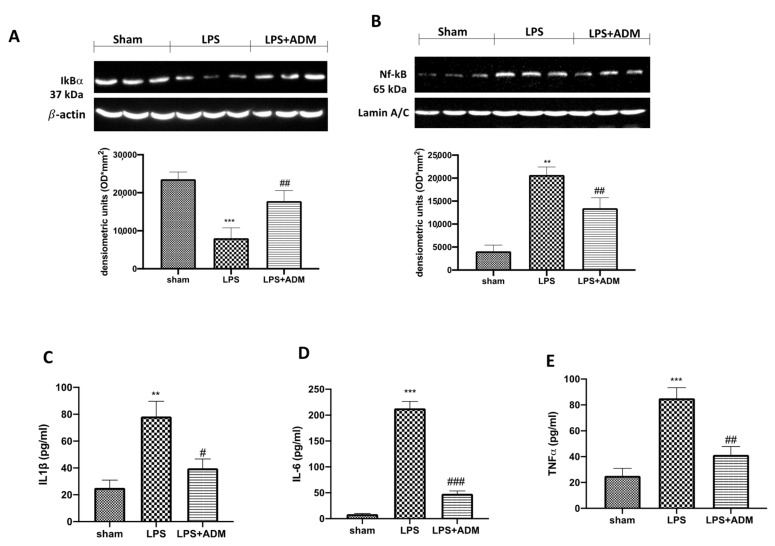
Western blot analysis for NF-κB (**B**) and IκB-α (**A**). Levels of inflammatory cytokine in lung tissues: IL-1β (**C**) IL-6 (**D**), TNF-α (**E**). A demonstrative blot of lysates for NF-kB and IκB-α with a densitometric analysis for all animals is shown. Data are expressed as the mean ± SEM of N = 6 mice/group. ** *p* < 0.01 vs. sham; *** *p* < 0.001 vs. sham; # *p*< 0.05 vs. LPS; ## *p* < 0.01 vs. LPS; ### *p* < 0.001 vs. LPS.

Additionally, we investigated one of the key inflammatory pathways involved in LPS-induced ALI, the NF-κB pathway. Our Western blot analysis showed a basal expression of IκB-α in Sham mice, while LPS i.t. instillation significantly decreased IκB-α expression in lung samples (Figure 8A). At the same time, nuclear NF-κB expression was significantly higher in LPS-treated animals compared to the sham group (Figure 8B). ADM treatment reduced IKB-α degradation and, consequently, nuclear translocation of NF-κB induced by LPS (Figure 8A,B). Additionally, to confirm the anti-inflammatory effect of ADM, we measured the levels of pro-inflammatory cytokines in lung tissues. We found that IL-1β (Figure 8C), IL-6 (Figure 8D) and TNF-α (Figure 8E) levels were markedly increased in the LPS group, compared to the sham mice. On the contrary, ADM was able to decrease the lung levels of these pro-inflammatory cytokines (Figure 8C–E).

## 4. Discussion

ALI is an acute inflammatory illness that can advance to a severe stage known as ARDS, which is marked by a high death rate. These clinical syndromes are characterized by a loss of barrier functionality by alveolar epithelial and pulmonary capillary endothelial cells, resulting in respiratory failure in critically ill individuals. To study the molecular mechanisms underlying ALI, the experimental endotoxin (bacterial LPS) model by intratracheal instillation in mice was used [7,66]. In experimental ALI, the lung parenchyma is damaged by the generation of a complex network of inflammatory cytokines and chemokine, including IL-1β, IL-6, and TNF-α [9]. Moreover, activation of oxidative stress with excessive release of ROS produced by activated pulmonary macrophages and transmigrated neutrophils in the interstitial and alveolar compartments has been demonstrated [66,67]. Then, an imbalance is created between the oxidant/antioxidant system, which, combined with the activated inflammatory response, causes diffuse alveolar damage with intrapulmonary hemorrhage, edema and fibrin deposition. Therefore, this study was designed to evaluate the effects of ADM in controlling the inflammatory and oxidative response in LPS-induced ALI. The anti-inflammatory and antioxidant properties of ADM, a member of the ALIAmide family, have been extensively demonstrated in previous studies [23,25,34,50,68,69]. First, histopathological investigation showed that ADM 2% aerosol administration significantly repaired the morphological and histological alterations in lung tissue, induced by LPS instillation. Extensive neutrophil infiltration, the large release of inflammatory mediators, an increase in capillary permeability, and severe interstitial edema are all thought to play important roles in the pathogenesis of ALI [70,71,72,73]. ADM 2% treatment was able to reduce all these parameters, as demonstrated by a reduction in MPO activity and levels of cell infiltration in BALF, as well as significant decrease in pro-inflammatory cytokines and lung edema. ALI and ARDS are often characterized by inappropriately/chronically activated MCs [74]. Many proteases, such as chymase and tryptase, are substances secreted by MCs activation, and contribute to inflammatory cells infiltration, cytokine production, and increased vascular permeability, exacerbating inflammation [75]. At this regard, the anti-inflammatory properties of ADM are mainly due to the control of MC activation and, as expected, our results confirmed a reduced number of MCs after ADM treatment. Consequently, we observed an important reduction in chymase and tryptase expression after ADM 2% administration, confirming the control of ADM on MCs activity. Additionally, it has also been demonstrated that ADM increased endogenous levels of antioxidant enzymes [25], indirectly modulating the NF-κB pathway. Indeed, ALI is characterized by excessive ROS production, causing imbalance to antioxidant system, and resulting in the release of substances modulating the endothelial dysfunction and disruption responsible for the principal clinical manifestations of the syndrome. ADM 2% aerosol administration also had positive results on endogenous levels of enzymes involved in oxidative stress; in fact, the treatment significantly counteracted the LPS-induced down-regulation of antioxidant indicators, as shown by the effect of CAT and GSH levels, as well as HO-1 and MnSOD expressions. These antioxidant enzymes are regulated by the Nrf2 pathway. Moreover, the functional crosstalk between Nrf2 and NF-κB is well known. The absence of Nrf2 is associated with increased oxidative stress, leading to an increase in cytokine production, as NF-κB is more readily activated in oxidative conditions [76,77]. Our Western blot analysis showed that ADM 2% was able to upregulate Nrf2 expression, responsible for antioxidant response, as well as to modulate the NF-κB pathway. To confirm the protective function of ADM, we also investigated the levels of proinflammatory cytokines in lung tissue. Again, ADM treatment significantly counteracted the LPS-induced increase in inflammatory mediator levels.

## 5. Conclusions

In conclusion, our data demonstrated that ADM 2% aerosol was able to reduce lung damage and cell infiltration, as well as the overexpression of proinflammatory cytokines. The protective effects of ADM 2% aerosol, probably due to the control of MC degranulation and the upregulation of endogenous antioxidant enzymes, modulate the inflammatory and oxidative response. Therefore, we suggest that ADM 2% aerosol can be considered as a potential candidate in the management of ALI.

## Data Availability

For a rule of our laboratory the datasets used in the current study are available from the corresponding author on reasonable request.
